# One in three adolescent schoolgirls in urban northwest Ethiopia is stunted

**DOI:** 10.1186/s13052-018-0459-z

**Published:** 2018-03-07

**Authors:** Samuel Mersha Birru, Aysheshim Kassahun Belew, Amare Tariku

**Affiliations:** 1 Gondar University Teaching and Referral Hospital, University of Gondar, Gondar, Ethiopia; 2 Department of Human Nutrition, Institute of Public Health, University of Gondar, Gondar, Ethiopia

**Keywords:** Stunting, Adolescent girls, Media exposure, Urban settlement, Ethiopia

## Abstract

**Background:**

Poor nutritional status of adolescent girls has a negative effect on the next generation as undernourished adolescents enter pregnancy with poor nutrient reserve. However, there is scarcity of evidence showing the burden of stunting among adolescent girls in Ethiopia. Therefore, the objective of this study aimed to assess prevalence of stunting and associated factors among school adolescent girls in Gondar City Administration, northwest Ethiopia.

**Methods:**

Cross-sectional study was conducted from March to April, 2017. A multi-stage sampling technique was used to select812 school adolescent girls. World Health Organization Anthro-plus software was used to analyze anthropometric data into Height for Age Z-score. A multivariable logistic regression analysis was employed to identify the factors associated with stunting. Adjusted Odds Ratio (AOR) with 95% confidence interval was used to show the strength of association, while a *P*-value< 0.05 of was used to declare the significance of association.

**Results:**

The overall prevalence of stunting among adolescent girls was 33.1% (95% CI: 29.9, 36.5). Middle age of adolescence (AOR = 0.22, 95% CI: 0.15, 0.34), unsatisfactory media exposure (AOR = 1.69, 95% CI: 1.01, 2.84) and poor mother’s education (AOR = 2.84, 95% CI: 1.07, 7.94) were significantly associated with stunting.

**Conclusions:**

One-third of adolescent girls are stunted in Gondar City which suggests the serious public health importance of the problem. Enhancing mother’s education and media exposure are critical to address the high burden of stunting.

## Background

Globally, adolescents comprise 16% (1.2 billion) of world population, more than 40% of which are adolescent girls. Of the total adolescent girls, 16 million give birth every year, off this 23% are from the sub-Saharan Africa, the region where the highest burden of maternal and child under nutrition is frequently reported [1]. Nutrition, optimal energy and micronutrient intake, plays an important role in supporting physical, mental and emotional development of adolescents [2, 3].

Poor nutritional status of adolescent girls, including stunting, has a negative effect on generation. Hence, undernourished adolescents enter pregnancy with poor nutrient reserve. Consequently they will give birth to low birth weight baby that is more vulnerable to chronic disease in later life due to early fetal programming [4, 5]. Moreover, it is associated with high risk of morbidity and mortality, delayed mental development, reduced intellectual capacity, poor educational achievement, school attendance and concentration [6, 7]. As a result, under nutrition is considered as a strong predictor of human capital and social progress [8]. However, not trivial numbers of adolescents are undernourished in developing countries [8–10]. The high prevalence of adolescent stunting is reported in Asia and Africa. As an illustration, nearly half of (47.0–47.4%) adolescent girls are stunted in India [11, 12] and Bangladesh (32–49%) [13, 14]. On the other hand, about 12.1 and 17.4% of adolescents have stunted growth in Kenya [15] and Nigeria [16], respectively, which is lower than Asian countries report. As like other developing countries, adolescent stunting existed as public health problem in Ethiopia [17]. One-third (31.5%) of adolescent girls are affected by stunting in Amhara region, the region where the study area is located [18]. Similarly, higher prevalence (25.5%) of stunting is shown in Eastern Tigray [19]. Nevertheless, a bit lower burden of adolescent stunting is reported in City Administration of the country: 15.6% in Adama [18] and 12.2% from Adawa city [19].

Some of the former literatures identified the determinants of adolescent stunting. Attending public schools, low maternal education [16], not starting menarche [20], being in the middle age range [21], rural residence [21], use of unimproved water sources [19] and larger family size [19] were elicited as significant predictors of stunting.

Even though adolescence is identified as a part of critical window to address different nutritional problems [8], adolescent nutrition receives less attention as compared to child and maternal nutrition issues [22]. Recently the government of Ethiopia starts to pay especial emphasis to enhance adolescent’s nutritional status: the revised National Nutrition Program (NNP) and national school health and feeding programs are some of the current efforts which has been implemented in the country at large [17, 23]. The NNP baseline survey report showed that 23% of adolescent girls are stunted in Ethiopia [24] which implies the critical importance of regular monitoring and evaluation of the magnitude of stunting. Also, the observed discrepancy in the burden of adolescent stunting between urban and rural settlements [18, 19, 25, 26], necessitates further, separate, investigation of the specific determinants of stunting, though only few literature is available on the specific area. Therefore, this research intended to assess prevalence and associated factors of stunting among school adolescent girls in Gondar City Administration, northwest Ethiopia.

## Methods

### Study setting and design

A school based cross-sectional study was conducted from March to April, 2017 in Gondar City Administration, northwest Ethiopia. Gondar City is a capital of North Gondar Zone, one of the densely populated Zone in Amhara National Regional State. The city has 11 rural kebeles (*the smallest administration unit in Ethiopia*) and 12 sub-cities. Based on the Central Statistics Agency population projection in 2014, a total of 306,246 people lives in the City [27]. In the City, 30 primary and 9 secondary and preparatory schools are owed by the government, while 12 primary and 5 secondary and preparatory are registered as private schools. At the moment, a total of 38,960 students in both public and private schools were found attending their education, of which 20,475 were female adolescent girls.

### Sample size and sampling procedure

All adolescent girls (10–19 years of age) attending governmental and private schools in Gondar City Administration were the source population for this study. The sample size was calculated using a single proportion formula by considering the following assumptions; 12.2% as previous prevalence of stunting among adolescent girls in Adwa Town in Tigray Region, North Ethiopia [19], the determination of sample size(n) for this study was calculated by the following formula.

$$ \mathrm{n}={\mathrm{z}}^2\mathrm{p}\left(1\hbox{-} \mathrm{p}\right)/{\mathrm{d}}^2 $$

Where, Z is critical value for normal distribution at 95% confidence level of two tailed which is equal to 1.96, P is the previous prevalence of stunting among adolescent girls was 12.2%, and d is degree of precision it was 3%. Then, multiply by the design effect of 1.5. With this assumption the sample size (n) was = (1.96) 0.122(1–0.122)/ (0.03) × 1.5 = 753. Then, 75 of respondents were obtained by adding 10% of non-response of the respondents. Finally, sample size computed was 812.

A multistage stratified sampling followed by systematic sampling technique was employed to select study participants. Primarily, secondary and preparatory schools were stratified into government and private. Then, eight governmental and three private schools were selected by lottery method. Sampling frame was prepared by taking a complete list of students enrolled in the selected schools. Quantity of students included in each school was proportional to the total eligible students. Following estimation of sampling fraction (k), samples were selected using systematic sampling technique.

### Data collection tool and procedures

Structured self-administered questionnaire was used to collect data. The questionnaire was first developed in English and translated into Amharic then back translated into English by an English language and public health expert to check the consistency. Pretest was done on 5 % of the sample out of the study area. Data collectors and supervisors were trained for 2 days about methods of interview and anthropometric measurements. A total of four clinical nurses as data collector and two public health experts as supervisor were recruited for the study. During the data collection, close supervision was done by the principal investigator and supervisors.

### Variable measurements

Height was measured to the nearest 0.1 cm using height measuring stadiometer in standing position with a sliding headpiece. The subject stood up on the basal part of the device with feet together. The shoulders, buttocks, calf and heels touched the vertical stand of the stadiometer. The adolescent girls stand with their eyes in the Frankfort horizontal plane. Considering the standardized criteria, stunting is defined as height –for- age (HAZ) value of less than two standard deviations from the WHO Growth reference standard (2007). Consequently, adolescents with HAZ of <− 2 were categorized as stunted, whereas those with HAZ of ≥ − 2 were considered as not stunted. On the other hand, adolescent those body mass index–for- age (BMI for age) < − 2 Z score were classified as wasted [28].

Dietary diversity of adolescents was measured using a standardized and validated tool containing 10 food groups. Using the participants verbal report, food items eaten by adolescents was categorized under respective food groups. Then if a girl ate five or more food groups, she was considered as having adequate dietary diversity, while those who consumed less than five food groups were deemed to have inadequate dietary diversity [29].

Concerning media exposure an adolescent who read a newspaper or magazine or listen to radio, or watched television at least once per week were considered as having satisfactory media exposure. Food security was assessed using 6-items and the sum of affirmative responses to the six questions was taken. The food security status of households labeled as 0 and 1 was described as food secure and food insecure, respectively [30].

The household wealth index was determined using Principal Component Analysis (PCA) by considering the household assets, such as quantity of cereal products, house, livestock and agricultural land ownership. First, variables were coded between 0 and 1. Then, the variables entered and analyzed using PCA, and those variables having a communality value of greater than 0.5 were used to produce factor scores. Finally, the factor scores were summed and ranked into first, second, third and fourth quartile.

### Data processing and analysis

All returned questionnaire were checked for completeness and consistency of responses manually. Then, the collected data were entered into EPI-INFO version 3.5.3 and exported to SPSS version 20 for further analysis. Also, WHO Anthro- plus software was used to generate HAZ and BMI for age there by to ascertain stunting and wasting of adolescents. Descriptive statistics, such as figures, tables and frequencies, were used to summarize variables. Both bivariable and multivariable binary logistic regression analyses were used to identify factors associated with stunting. Variables with *p*-value less than 0.2 in the bivariable analysis were fitted into the multivariable logistic regression analysis. Both Crude Odds Ratio (COR) and Adjusted Odds Ratio (AOR) with the corresponding 95% Confidence Interval were calculated to show the strength of association. Finally, in the multivariable analysis, variables with a *P*-value of less than 0.05 were considered as statistically significant.

## Results

A total of 812 adolescent girls were included in the study with a response rate of 97.5%. The median age of the adolescents was 16 with (Inter-Quartile Range (IQR) of 14–17 years. Substantial proportion, 742 (93.3%) of participants resided in urban kebeles. More than half of the mothers had informal education 437(55%). Nearly half, 365(45.9%) of households had five and above family size and 168(21.1%) were found in the rich wealth category (Table 1).

**Table 1:** Socio-demographic characteristics of school adolescent girls and their parents, Gondar City Administration, northwest Ethiopia, 2017 (*n* = 795)

Variables	Frequency	Percent
Age		
Early	140	17.6
Middle	415	52.2
Late	240	30.2
Level of education		
Primary	301	37.6
High school	428	53.8
Preparatory school	66	8.3
Religion		
Orthodox	760	95.6
Muslim	28	3.5
Others	7	0.9
Place of residence		
Urban	742	93.3
Rural	53	6.7
Ethnicity		
Amhara	754	94.8
Oromo	12	1.5
Tigray	9	1.1
Others	20	2.5
Marital status of girls		
Not Married	713	89.7
married	82	10.3
Educational status of father		
Informal education	379	47.7
Primary	100	12.6
Secondary	131	16.5
College and above	185	23.3
Occupation of father		
Government employee	259	32.6
Farmer	208	26.2
Daily laborer	40	5
Merchant	161	20.3
NGOS	45	5.7
Educational status of mother		
Informal education	437	55
Primary	122	15.3
Secondary	128	16.1
College and above	108	13.6
Occupation of mother		
Government employee	129	16.2
Housewife	499	62.8
Daily laborer	41	5.2
Merchant	85	10.7
Others	41	5.2
Family size		
< =5	430	54.1
> 5	365	45.9
School enrolled		
Government	630	79.2
Private	165	20.8
Wealth index		
1st quintile	168	21.1
2nd quintile	219	27.5
3rd quintile	208	26.2
4th quintile	200	25.2

Of the total respondents, 631(79.4%) had satisfactory media exposure. Large proportion 776(97.6%) of participants consumed grains and roots and 600(75.5%) of had adequate dietary diversity. Two-third 499(62.8%) of the households were food secured (Table 2). Availability of home gardening was reported in 131(16.5%) of the households, but 52.7% of which used the product for sale. Majority, 620(79.1%) also had home latrine and used improved drinking water 783(98.5%) (Table 3).

**Table 2:** Nutritional and health related characteristics of school adolescent girls, Gondar City Administration, northwest Ethiopia, 2017 (*n* = 795)

Variables	Frequency	Percent
Meal frequency per day		
< 3	87	10.9
> =3	708	89.1
Household food security		
Secured	499	62.8
In secured	296	37.2
Dietary diversity score		
Adequate	600	75.5
Inadequate	195	24.5
Two weeks history of illness		
Yes	160	20.1
No	635	79.9
Member of association		
Yes	148	18.6
No	647	81.4
Media exposure		
Satisfactory	631	79.4
Unsatisfactory	164	20.6
Menstruation status		
Yes	599	71.3
No	196	24.7
Age of first menarche		
10–13	163	20.5
14–16	358	45
17–19	78	5.8
Past history of pregnancy		
Yes	35	4.4
No	760	95.6

**Table 3:** Environmental characteristics of school adolescent girls in Gondar City Administration, Northwest Ethiopia, 2017

Variables	Frequency	Percent
Availability of home gardening		
Yes	131	16.5
No	664	83.5
Purpose of home gardening		
For home	13	9.9
For sale	69	52.7
For sale and home	49	37.4
Source of drinking water		
Improved	783	98.5
Unimproved	12	1.5
Wate water treatment		
yes	595	74.8
No	200	25.2
Distance of water source		
< =30 min	785	98.5
> 30	12	1.5
Availability of home latrine		
yes	629	79.1
No	166	20.9
Availability of waste disposal/garbage		
Yes	735	92.5
No	60	7.5
Hand washing after toilet		
Yes	784	98.4
No	11	1.4
Use of detergent/soap		
Yes	702	88.3
No	93	11.2

**Fig. 1:**
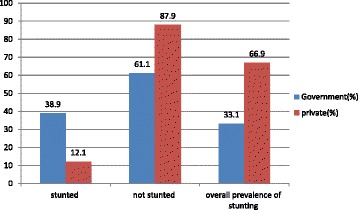
Prevalence and distribution of stunting among adolescent girls stratified by school type, Gondar City Administration, northwest Ethiopia, 2017

The study noted that the overall prevalence of stunting (HAZ < − 2) was 33.1% and about 2.4%of adolescent girls were wasted. The multivariable logistic regression analysis showed that age of the respondent, unsatisfactory media exposure and educational status of mothers were significantly associated with stunting (Fig. 1).

Adolescent girls found in the early and middle age range were found at 74% (AOR = 0.26, 95% CI: 0.00, 0.22) and 78% (AOR = 0.22, 95% CI: 0.15, 0.34) lesser odds of stunting compared to late adolescent girls. The odds of stunting were higher among adolescents who had unsatisfactory media exposure (AOR = 1.69, 95% CI: 1.01, 2.84) and whose mothers completed college and above education (AOR = 2.84, 95% CI: 1.02, 7.94) (Table 4).

**Table 4:** Bivariate and multivariable logistic regression output showing that factors associatedwith stunting among school adolescent girls, Gondar City Administration, northwest Ethiopia, 2017

Variables	Stunting		Crude Odds Ratio with 95% C)	Adjusted Odds Ratio with 95% CI
	Stunted	Not stunted		
Age of adolescent				
Early	3(2.1%)	137(97.9%)	0.01(0.00,0.04)	0.26(0.00,0.22)^a^
Middle	112(27. %)	303(73%)	0.23(0.16,0.32)	0.22(0.15,0.34)^a^
Late	148(61.7%)	92(38.3%)	1	1
Educational level of respondents				
Primary	48(16%)	253(84%)	0.15(0.08,0.26)	0.68(0.28,1.85)
High school	178(41.6%)	250(58.4%)	0.56(.33,.94)	0.95(0.49,1.85)
Preparatory school	37(56.1%)	29(43.9%)	1	1
School enrolled				
Government	243(38.9%)	382(61.1%)	4.61(2.77,7.46)	1.49(0.71,3.14)
Private	20(12.1%)	145(87.9%)	1	1
Educational status of father				
Informal education	156(41.2%)	223(58.8%)	2.11(1.43,3.12)	0.91(0.51,1.74)
Primary	29(29%)	71(71%)	1.23(0.71,2.13)	0.71(0.32,1.54)
Secondary	32(24.4%)	99(75.6%)	0.97(.58,1.64)	0.66(0.32,1.36)
College and above	46(24.9%)	139(75.1%)	1	1
Educational status of mothers				
Informal education	178(40.7%)	259(59.3%)	2.85(1.70,4.76)	2.84(1.02,7.94)^a^
Primary	29(23.8%)	93(76.2%)	1.29(0.69,2.43)	2.01(0.69,5.81)
Secondary	35(27.3%)	93(72.7%)	1.59(0.48,2.88)	2.26(0.85,6.03)
College and above	21(19.4%)	87(80.6%)	1	1
Occupation of the mothers				
Government employee	44(34.1%)	85(65.9%)	1.61(0.72,3.57)	1.66(0.55,5.03)
Housewife	175(35.1%)	324(64.9%)	1.67(0.80,3.51)	0.78(0.30,2.04)
Daily laborer	15(36.6%)	26(63.4%)	1.79(0.69,4.65)	0.63(0.18,2.22)
Merchant	19(22.4%)	66(77.6%)	0.89(0.37,2.14	0.41(0.13,1.28)
Others	10(24.4%)	31(75.6%)	1	1
Dietary diversity				
Adequate	187(31.2%)	413(68.8%)	1	1
Inadequate	76(39%)	119(61%)	1.41(1.00,1.97)	1.06(0.70,1.61)
Wealth index				
1st quintile	77(45.8%)	91(54.2%)	2.23(1.44,3.44)	1.72(0.95,3.12)
2nd quintile	73(33.3%)	146(66.7%)	1.31(0.86.1.57)	1.01(0.61,1.86)
3rd quintile	58(%)	150(%)	1.01(0.66,1.57)	0.82(0.47,1.43)
4th quintile	55(27.9%)	145(72.1%)	1	1
Availability of latrine				
Yes	192(30.5%)	437(69.5%)	1	1
No	71(42.8%)	95(57.2%)	1.70(1.19,2.41)	0.74(0.38,1.45)
Menstruation status				
10–13	57(35%)	106(65%)	0.40(0.09.1.87)	0.86(0.54,1.37)
14–16	152(42.5%)	206(57.5%)	0.55(0.12,2.51)	0.99(0.17,5.61)
17–19	4(57.1%)	3(42.9%)	1	1
Media exposure				
Satisfactory	225(35.7%)	406(64.3%)	1	1
Unsatisfactory	38(23.2%)	126(76.8%)	0.54(0.36,0.81)	1.69(1.01,2.84)^a^
Past history of pregnancy				
No	222(39.3%)	343(60.7%)	1	1
Yes	19(54.3%)	16(45.7%)	1.84(0.92,3.64)	0.46(0.21,0.98)
Past two weeks history of illness				
No	222(39.3%)	343(60.7%)	1	1
Yes	19(3.7%)	501(96.3%)	0.72(0.49,1.06)	0.51(0.23,1.50)
Household food security				
Secured	174(34.9%)	325(65.1%)	1	1
In secured	89(30.1%)	207(69.9%)	1.25(0.91,1.71)	0.81(0.52,1.26)
Availability of waste disposal/garbage				
No	29(48.3%)	31(51.7%)	2.00(1.18,3.40)	0.73(.371,1.43)
Yes	234(31.8%)	501(68.2%)	1	1
Availability of home gardening				
No	213(32.1%)	451(67.9%)	0.77(0.52,1.13)	0.69(0.42,1.12)
Yes	50(38.2%)	81(61.8%)	1	1

^a^indicate significant at *p* value less than 0.05 in multivariable logistic analysis

## Discussion

This study illustrated that one of the three (33.1%(95% CI (29.9, 36.5)) adolescent girls were stunted, suggesting the issue as one of the major public health problem. The finding is consistent with the previous report in Nepal 32% [31]. However, the report is lower than the former Asian and African countries investigations, for instance.

51.9% of adolescent girls in Assum, India [11] and 47% in panangal [12], districts of India, are stunted. Similarly, the high magnitude of adolescent stunting is noted in Bangladesh 49% [14] and Nigeria 57.8%. The high prevalence of stunting in India [11] could be attributed to the nature of the study setting where only adolescents from the slum areas were included, while our study considered random selection of samples with regard to place of residence. Obviously, slum areas are well-known with insanitary living environments and lower socio-economic status, underlying causes of under nutrition, compared to non-slum settlements [32]. On the other hand, half participants in Bangladesh were pregnant compared to 95.6% of samples had no history of pregnancy in the current study. This could explain the increased magnitude of stunting Bangladesh. In fact, pregnancy demands significantly high energy and micronutrients even the requirement increases in case of adolescence pregnancy to support tremendous growth of both the adolescent and the fetus. Therefore, adolescence pregnancy amplifies the vulnerability of girls for under nutrition, including stunting [33]. In contrast, our finding is higher than the former local and abroad reports, such as Tigray Region, 26.5% [34], Adwa Town 12.2% [19], western Kenya 12.1% [15] and Nigeria 17.9%. This discrepancy could be related to socio-economic disparities between the study settings.

The result of the adjusted analysis showed that girls in the early and middle adolescence were 74% and 78% times less likely to be stunted, respectively, as compared to late adolescent girls. Similar finding was reported in urban set up of Kathmandu valley [31]. This could be due to the fact that sudden increase in height after the first menstruation in the middle age group and in the later time. Therefore, enhancing energy and micronutrient intake has special importance in the late adolescence.

Adolescent girls whose mothers did not have formal education were 2.84 times more likely to be stunted as compared to those whose mothers completed college and above education. This finding is supported by the study done in Bangladesh [14] and Nigeria. This may due to the fact that low job access and poor household decision making power are common social problems of poorly educated mothers which could affect nutritional status of children, including adolescents. Furthermore, early marriage and poor utilization of family planning are also observed among women with low education [35].

The odds of stunting were 1.69 times higher among adolescent girls who had unsatisfactory media exposure compared to those who had satisfactory media exposure. The observed association could be explained by the positive effect of media in changing unhealthy eating behaviors of the community which in turn could contribute for better nutritional outcomes through delivering reliable and clear nutrition information [25]. Moreover, adolescent girls with adequate media exposure might indirectly show their good economic status which in turn indicates resource availability at the household level to improve purchase diversified diet. One the previous scientific report also affirmed that poor feeding practice was associated with adolescents limited media exposure which in turn affect their nutrition condition [36].

Though this study tried fill the knowledge gap by showing the burden and related characteristics of adolescent stunting, some of the limitations should be taken into consideration. Firstly, water and sanitary services of the school environment is not assessed. Secondly, recall bias in measuring the dietary diversity and household food security status are also the possible limitation of the study, although different efforts, as described in the method section, were made to minimize it.

## Conclusion

In summary, one-third of adolescent girls are stunted in Gondar District which confirmed serious public health importance of the problem. Age and media exposure of adolescents and mothers education were significantly associated with stunting. Therefore enhancing mother’s education and access to media are important to address the high burden of stunting.

## Abbreviations

AOR: Adjusted odds ratio
BSC: Bachelors of science
CI: Confidence interval
CM: Centimeter
COR: Crud odds ratio
DDS: Dietary diversity score
EDHS: Ethiopian demographic and health survey
FAO: Food and Agriculture Organization
HAZ: Height -for-age
HFS: Household food security
MSC: Masters of science
PCA: Principal component analysis
SPSS: Statistical package for social sciences
SRS: Systematic random sampling
WHO: World Health Organization
